# NOX4-dependent ROS production by stromal mammary cells modulates epithelial MCF-7 cell migration

**DOI:** 10.1038/sj.bjc.6605847

**Published:** 2010-08-17

**Authors:** N Tobar, J Guerrero, P C Smith, J Martínez

**Affiliations:** 1 Cellular and Molecular Biology Laboratory, INTA, Universidad de Chile Casilla 138, Santiago 11, Chile; 2 Laboratory of Periodontal Physiology, Dentistry Academic Unit, Faculty of Medicine, Pontificia Universidad Católica de Chile, Santiago, Chile

**Keywords:** NOX4, ROS, migration, breast cancer, stroma

## Abstract

**Background:**

The influence of the stromal microenvironment on the progression of epithelial cancers has been demonstrated. Unravelling the mechanisms by which stromal cells affect epithelial behaviour will contribute in understanding cellular malignancy. It has been proposed that redox environment has a role in the acquisition of malignancy. In this work, we studied the influence of epithelial cells on the stromal redox status and the consequence of this phenomenon on MCF-7 cell motility.

**Methods:**

We analysed in a co-culture system, the effect of RMF-EG mammary stromal cells on the migratory capacity of MCF-7 cell line. To test whether the NOX-dependent stromal redox environment influences the epithelial migratory behaviour, we knocked down the expression of NOX4 using siRNA strategy. The effect of TGF-*β*1 on NOX4 expression and activity was analysed by qPCR, and intracellular ROS production was measured by a fluorescent method.

**Results:**

Migration of MCF-7 breast epithelial cells was stimulated when co-cultured with RMF-EG cells. This effect depends on stromal NOX4 expression that, in turn, is enhanced by epithelial soluble factors. Pre-treatment of stromal cells with TGF-*β*1 enhanced this migratory stimulus by elevating NOX4 expression and intracellular ROS production. TGF-*β*1 seems to be a major component of the epithelial soluble factors that stimulate NOX4 expression.

**Conclusions:**

Our results have identified that an increased stromal oxidative status, mainly provided by an elevated NOX4 expression, is a permissive element in the acquisition of epithelial migratory properties. The capacity of stromal cells to modify their intracellular ROS production, and accordingly, to increase epithelial motility, seems to depend on epithelial soluble factors among which TGF-*β*1 have a decisive role.

Convincing experimental data support the involvement of cellular stroma in carcinoma progression (De Wever and Mareel, 2003). Particularly, there is strong evidence supporting the role of the stromal cells in breast cancer growth and development (Radisky and Radisky, 2007). Available data suggest that carcinoma and stromal cells maintain an active dialogue with functionally reciprocal consequences. In that way, soluble stromal products are able to modify the invasive potential of carcinoma cells, and epithelial factors are responsible for the development of a ‘stromal response’ that in some tumours, such as those of breast, stomach and pancreas, is termed desmoplasia (Elenbaas and Weinberg, 2001).

Among the variety of cell types that constitute the tumoral microenvironment, fibroblasts have been mentioned as an important component. Under the stimuli of tumour cells, fibroblasts are able to acquire some functional properties that confer them a ‘reactive’ phenotype, becoming what is termed as carcinoma-associated fibroblasts (CAFs) (Egeblad *et al*, 2005). Considerable experimental work sustains that CAFs produce tumour-promoting and angiogenic factors along with chemokines that contribute to the recruitment of bone marrow-derived cells (Ostman and Augsten, 2009).

An emerging issue in the carcinogenesis field has arisen from studies that revealed that a chronic inflammatory condition predisposes the epithelial component of tissues to develop a tumour (Coussens and Werb, 2002). A key element in this process is the establishment of an oxidative stress at the tissue level that generates an increase in reactive oxygen species (ROS) phenomenon that, as has been previously demonstrated, contributes to carcinoma malignancy (Blanchetot and Boonstra, 2008). Some previous observations indicate that ROS, acting as secondary messengers, have a role in the regulation of cell proliferation, which is crucial in tumour cells that produce elevated ROS levels (Szatrowski and Nathan, 1991).

The cellular oxidative stress that contributes to the aetiology of cancer is produced by an endogenous generation of ROS that arises from two main sources: the mitochondria and the NAD(P)H oxidase (NOX) system (Dröge, 2002). In non-phagocytic cells, the NOX family is a key component of the so-called ‘redox signalling system’ that regulates many cellular responses by modulating the intracellular ROS content. The alteration in the cellular oxidative status generates changes in processes such as cell migration and growth (Ushio-Fukai and Nakamura, 2008). For example, it has been demonstrated that the NOX-dependent basal oxidative stress of both prostatic cell lines and normal epithelial cells is directly associated with the expression of the aggressive phenotype (Kumar *et al*, 2008). On the other hand, it has also been demonstrated that NOX1 stimulates cell proliferation, activating the cell cycle by reducing the requirement for growth factors to maintain the expression of cyclin D1 and stimulating the transcriptional activation of Fos family genes during the immediate early gene response (Ranjan *et al*, 2006).

In this study we investigate the influence that a human mammary fibroblast cell line (RMF-EG cells) exerts on the migratory behaviour of the weakly invasive human mammary cell line MCF-7. Using a co-culture system as a model of stromal–epithelial interaction, we analysed the role of the NOX-dependent oxidative intracellular status of RMF-EG cells on the migration of MCF-7 cells. In addition, we studied the role of TGF-*β*1, a growth factor that is highly expressed in tumour cells, in the maintenance of a pro-oxidative environment and the consequent production of soluble factors that stimulate epithelial migration.

## Materials and methods

### Chemicals reagents

Human recombinant TGF*β*1 was purchased from R&D Systems (Minneapolis, MN, USA). 2′,7′-dichlorodihydrofluorescein diacetate (H_2_DCFDA), *N*-acetylcysteine (NAC), diphenyleneiodonium chloride (DPI) and bovine catalase were from Sigma (St Louis, MO, USA). SB431542 inhibitor was from TOCRIS Bioscience (Business Park Ellisville, MO, USA).

### Cell culture and transfection methods

Human mammary cell lines MCF-7, MCF-10 and MDA MB-231 were obtained from the ATCC (Manassas, VA, USA) and were cultured in DMEM/F12 medium (Invitrogen, Carlsbad, CA, USA) supplemented with 10% fetal bovine serum (FBS) (Hyclone, Logan, UT, USA) and maintained in a humidified atmosphere of 37°C, 5% CO_2_. RMF-EG stromal cells were kindly provided by Dr Charlotte Kuperwasser (Tufts University, Boston, MA, USA) and cultured in DMEM medium (Invitrogen) supplemented with 10% FBS (Kuperwasser *et al*, 2004). MCF7 cells were transiently transfected with 2 *μ*g ml^–1^ of the plasmid encoding for the dominant-negative form of TGF-*β* receptor I (dnT*β*RI) or pcDNA3.1 (empty vector), using Lipofectamine 2000 reagent according to the manufacturer's protocol (Invitrogen). The 48 h post-transfected cells were used to stimulate migration of MCF-7 cells.

### Cell migration assay

MCF-7 and MCF-10 cell migration was studied using a 6.5-mm Transwell chamber with a pore size of 8 *μ*m (Corning, Corning, NY, USA). The Transwell membranes were coated with 10% FBS in culture media for 2 h at 37°C only on the underside. MCF-7 and MCF-10 cells (6 × 10^4^) were re-suspended in serum-free medium and seeded on the upper compartment of the chamber. RMF-EG cells that were untreated or pre-treated with DPI or NAC were used to induce migration of MCF-7 cells. For this, 4 × 10^4^ RMF-EG cells, suspended in culture media enriched with 1% FBS, were incubated with 5 *μ*M of DPI or 1 mM NAC for 16 h and were then washed with 1 × PBS twice, before placing the insert with migrating MCF-7 cells. Additionally, we evaluated the migratory ability of MCF-7 cells that were stimulated by RMF-EG cells pre-treated 16 h with 10 ng ml^–1^ of TGF-*β*1 in the presence or absence of 5 *μ*M of DPI (added 30 min before) and RMF-EG cells transfected with the dominant-negative version of dnT*β*RI. In this case, the lower chamber of the Transwell was filled with medium enriched with 1% FBS. Migration was allowed to occur for 24 h, after which cells of the upper membrane surface were removed by a cotton swab and repeatedly washed with PBS. In migration experiments performed in the presence of catalase, 3000 IU of bovine catalase (Sigma) was added to the lower chamber of the Transwell. Migration values were determined by counting five ( × 20) fields per chamber after fixing the membrane in methanol and staining the migratory cells on the lower side of the membrane with 0.2% crystal violet (Tobar *et al*, 2008).

### Co-culture in non-migrating conditions

We also performed Transwell experiments using a 0.4-*μ*m pore membrane (24 mm diameter) bicameral system that prevents cell migration. RMF-EG cells (5 × 10^5^) were cultured in the lower well and MCF-7 cells (5 × 10^5^) in the upper chamber of the Transwell in serum-free media for 24 h. Both cell types were lysed with TRIzol to extract RNA.

### Knockdown assays

For transient siRNA transfection, RMF-EG cells at 70% confluence were transfected using TransIT-siQuest (Mirus, Madison, WI, USA) at 1 : 600 dilutions in complete medium, according to the manufacturer's recommendation, with a final siRNA concentration of 50 nM for 72 h. The oligos used to inhibit NOX4 expression were obtained from Santa Cruz (sc-41586, Santa Cruz, CA, USA), which is referred in the text as siRNA NOX4-I, and from Integrated DNA Technologies (Coralville, IA, USA) with the following sequences: 5′-GCCUCUACAUAUGCAAUAA-3′ and is referred to as NOX4-II (Carmona-Cuenca *et al*, 2008). As a control unsilenced siRNA, the sc 37007 oligo from Santa Cruz was used.

### Measurement of intracellular redox state

The oxidation-sensitive fluorescent probe H_2_DCFDA was used to analyse the total intracellular content of ROS. In a representative assay, RMF-EG cells were incubated with 5.0 *μ*M H_2_DCFDA in serum- and phenol red-free medium (Gibco Invitrogene, Carlsbad, CA, USA) for 30 min at 37°C. Cells were then washed and lysed with 0.1 N NaOH, and fluorescence was monitored using a microplate fluorometer (Spectra MAX, Gemini EM; Molecular Devices, Silicon Valley, CA, USA) with wavelengths of 480 and 530 nm for excitation and emission, respectively (Benhar *et al*, 2001). In experiments in which NOX-dependent ROS production was blocked, cells were transfected with NOX4 siRNA 72 h previous to ROS determination. In experiments in which the intracellular content of ROS was exogenously modulated, RMF-EG cells were pre-treated 24 h with 1–10 ng ml^–1^ of TGF-*β*1 before fluorometric determination of ROS.

### Immunodetection of NOX4

Serum-starved RMF-EG cells (10^6^ cells per dish) were treated with TGF-*β*1 (1–10 ng ml^–1^) for 24 h or transfected with siRNA for NOX4. Afterwards, total protein extracts were obtained using a lysis buffer containing 30 mM Tris-HCl pH 7.5, 5.0 mM EDTA, 150 mM NaCl, 1% Triton X-100, 0.5% sodium deoxycolate, 0.1% SDS and 10% glycerol, 2 mM PMSF, 2 *μ*g ml^–1^ pepstatin, 2 *μ*g ml^–1^ leupeptin and 1 mM orthovanadate at 4°C. Pellets were incubated for 1 h in lysis buffer at 4°C, sonicated for 5 s, boiled for 5 min and centrifuged at 16 500 **g** for 10 min at 4°C. The antibodies used were: mouse anti-*β-*actin (clone AC-15; Sigma) and anti-NOX4 rabbit polyclonal antibody (ab 41886; Abcam, Cambridge, MA, USA). Equal amounts of protein from different treatments were resolved by 10% SDS–PAGE and analysed by immunoblotting using the ECL chemiluminiscence detection kit (Amersham, Arlington Heights, FL, USA).

### Analysis of gene expression

Total RNA was isolated with Trizol (GIBCO, Carlsbad, CA, USA) from RMF-EG and control cells. Complementary DNA was generated by M-MLV reverse transcriptase (Promega, Madison, WI, USA) using oligo (dT) (Promega) as primer and 1.5 *μ*g of total RNA. Semiquantitative PCR reactions were performed using the following human-specific primers:

NOX1, forward 5′-GTACAAATTCCAGTGTGCAGACCAC-3′ and reverse 5′-CAGACTGGAATATCGGTGACAGCA-3′, PCR product size 397 and 250 bp, corresponding to the large and short forms of the molecule; NOX2, forward 5′-GTCACACCCTTCGCATCCATTCTCAAGTCAGT-3′ and reverse 5′CTGAGACTCATCCCAGCCAGTGAGGTAG-3′, PCR product size 225 bp; NOX4, forward 5′-TAGATACCCACCCTCCC-3′ and reverse 5′-GACTTATGACCGAAATGA-3′, PCR product size 294 bp; NOX5, forward 5′-ATCAAGCGGCCCCCTTTTTTCAC-3′ and reverse 5′-CTCATTGTCACACTCCTCGACAGC-3′, PCR product size 239 bp; *α*-Sma, forward 5′-GCAGCCCAGCCAAGCACTGTCAGGAAT-3′ and reverse 5′-AGCCCAGAGCCATTGTCACACACCAAGG-3′, PCR product size 674 bp; QNOX4, forward 5′-TAGATACCCACCCTCCCG-3′ and reverse 5′-TGGGCTCTTCCATACAAATC-3′, QPCR product size: 168 bp; 18S, forward 5′-GGACACGGACAGGATTGACA-3′ and reverse 5′-GGACATCTAAGGGCATCACAG-3′, PCR product size 240 bp.

The expression of 18S was analysed as a loading control. PCR products were subjected to electrophoresis on a 1.5% agarose gel, and DNA was visualised by ethidium bromide staining. Quantification of bands was determined using KODAK Molecular Imaging Software Version Upgrade 4.0 (Kodak, Rochester, NY, USA). To identify the expression of the different isoforms of NOX in RMF-EG cells, RT–PCR was run for 38 cycles. To test the effect of epithelial factors on NOX4 and NOX5 expression in co-culture, RT–PCR was performed at the linear zone of the saturation curve, that is, 32 cycles for both isoforms.

Quantitative PCR was performed with the real-time PCR system, LightCycler (Roche, Basel, Switzerland). Each reaction was conducted in glass capillaries with 50 ng of cDNA, in a final volume of 10 *μ*l. The PCR mixture contained LC FastStart DNA Master SYBR Green I (Roche Diagnostics, Basel, Switzerland), 3 mM MgCl_2_ and 0.3 pmol of each primer (forward and reverse). Fluorescence was analysed using LightCycler Analysis Software. The crossing point for each reaction was determined using the Fit point algorithm and manual baseline adjustment. Quantification was determined using a relative standard curve method with different amounts of a reference standard DNA generated from PCR amplicon for each target gene and expressed as copies per *μ*l. The template for the gene-specific standard curve was generated via conventional PCR and purification was performed using a Concert Rapid Gel extraction system (Gibco Invitrogene). The amount of transcript for each gene of interest was normalised against the 18S transcripts. The experiments were performed three times and in duplicate from three different RNA samples.

## Results

### Co-culture of MCF-7 cells with RMF-EG stromal cells enhanced their migratory activity: a possible role for NOX

**Figure 1:**
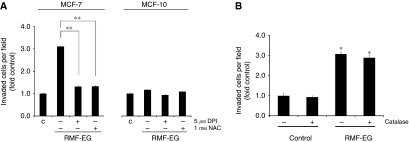
Mammary stromal cell line RMF-EG stimulates MCF-7 cell line migration. A possible role for ROS. (**A**) Co-culture system of epithelial and mammary stromal cells. RMF-EG (4 × 10^4^), MCF-7 (6 × 10^4^) and MCF-10 (6 × 10^4^) cells were seeded separately in the lower well and upper chamber, respectively, of a Transwell culture system. A group of RMF-EG cells were pre-treated for 16 h with 5 *μ*M diphenyleneiodonium chloride (DPI) or 1 mM NAC and washed before the introduction of the insert containing MCF-7 cells. (**B**) In the same experimental setting, migration of MCF-7 cells was evaluated in the presence or the absence of 3000 IU of catalase diluted in the lower compartment of the Transwell. Migration was evaluated after 24 h of co-culture by counting stained migrating cells on the lower side of the filter, as indicated in the Materials and Methods. Data represent the mean±s.e. of four independent experiments. Student's *t*-test was used to compare MCF-7 migrating cells co-cultured with RMF-EG *vs* control non-co-cultured cells and MCF-7 migrating cells co-cultured with DPI-pretreated RMF-EG cells. ^*^P<0.05, ^**^P<0.01.

To evaluate the inductive role of RMF-EG mammary stromal cells on MCF-7 migration, we established an 8-*μ*m pore size Transwell system, plating 4 × 10^4^ RMF-EG cells in the lower chamber surface and 6 × 10^4^ MCF-7 in the insert, according to the protocol described in the Materials and Methods. To analyse the involvement of stromal NOX-dependent ROS production on MCF-7 cells migration, we pre-incubated RMF-EG monolayer overnight with 5 *μ*M of the flavoprotein inhibitor DPI. To analyse the participation of ROS, independent of its intracellular source, we pre-incubated another group of RMF-EG stromal cells with 1 mM NAC, a potent antioxidant molecule with a broad spectrum of action. After this, cells were washed and the insert containing MCF-7 cells was installed, thus initiating the migration assay. As Figure 1A shows, co-culture of epithelial cells with intact stromal cells induced a three-fold increase in cell migration. Pre-treatment of RMF-EG cells with DPI or NAC abrogated this effect almost completely. To assess the specificity of the stromal effect on tumour cells, we performed a migratory assay in the same experimental conditions described above, but using the MCF-10 cell line, a non-tumour homologue of MCF-7 cells line. As Figure 1A shows, migration of MCF-10 cells was not modified by co-culture conditions or the presence of stromal cells that were pre-treated with antioxidant molecules. From the results on MCF-7 cells, two possible explanations arise: on one hand, under the stimulus of soluble epithelial factors, RMF-EG cells generate a soluble form of ROS that stimulates migration of MCF-7 cells and , on the other hand, the epithelial-induced changes in stromal redox status induces the expression of an unknown soluble factor that modulates MCF-7 migration. To evaluate these two possibilities, we performed migration assays in which 3000 IU of bovine catalase was included in the lower well of the Transwell. As Figure 1B shows, either in the presence or in the absence of RMF-EG cells, exogenous catalase does not affect the basal or the stimulated MCF-7 migration, suggesting that the generation of a redox-dependent migratory soluble factor from RMF-EG origin is the more plausible possibility.

### Expression of mRNA for NOX4 in stromal cells is stimulated by co-culture with MCF-7 cells

**Figure 2:**
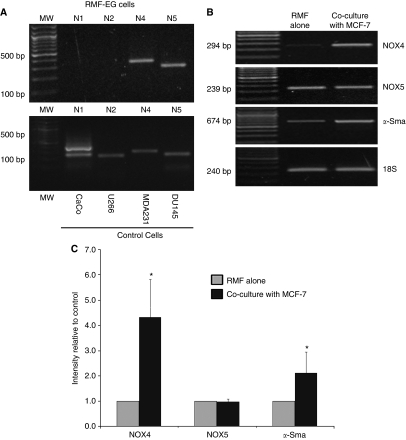
Soluble factors produced by MCF-7 cells induced the expression of NOX4 isoforms in RMF-EG cells. (**A**) The analysis of the expression of NOX family components in RMF-EG cells was performed by RT–PCR, as described in the Materials and Methods. Total RNA was prepared using TRIzol reagent and RT–PCR was performed using specific primers for human NOX 1, 2, 4 and 5 isoforms. PCR products were compared with those from an equal amount of mRNA from cell lines that express the different forms of NOX as CaCo cells (for NOX1), U266 (for NOX2), MDA 231 (for NOX4) and DU145 (for NOX5). All isoforms were analysed at 38 cycles. (**B**) Using a bicameral co-culture system similar to those used in experiments in Figure 1, with a membrane pore of 0.4 *μ*m to avoid cell migration, we cultured RMF-EG cells (in the lower well) and MCF-7 cells in the upper chamber of the Transwell system for 24 h in FBS-free culture medium. NOX4, NOX5 and *α*-Sma mRNA expression in RMF-EG cells were measured in a TRIzol-generated total mRNA solution as described in the Materials and Methods. NOX4 and NOX5 were analysed at 32 cycles and *α*-Sma at 24 cycles. Data in (**C**) represent the mean±s.e. of densitometric analysis of three different experiments. Student's *t*-test was used to compare control non-co-cultured cells *vs* co-cultured cells. ^*^P<0.05.

The ability of DPI to inhibit the RMF-EG-dependent MCF-7 cell migration strongly suggests that some of the isoforms described for NOX in these stromal cells were having a role is this paracrine stimulus. Therefore, using specific primers, we analysed which isoforms of the NOX system are expressed in RMF-EG cells. RT–PCR analysis of mRNA extracted from these cells shows that they expressed predominantly the NOX4 and NOX5 isoforms (Figure 2A). As a primer control, mRNAs from different cell lines that express specific NOX were used. NOX3 was not included in this analysis because it has been demonstrated that it is expressed mainly in fetal tissues (Cheng *et al*, 2001).

Once we identified that NOX4 and NOX5 were the predominant isoforms for NOX in RMF-EG cells, we analysed whether the expression of mRNA for these isoforms was modified by soluble factors generated by MCF-7 cells. To test this, RMF-EG cells were seeded in Transwell co-culture inserts (impermeable to cell migration) and co-cultured for the same period used in the migratory assay (24 h). In addition, to assess if the contact with mammary cell-produced factors constitutes a stimulus for myofibroblast differentiation, we analysed in RMF-EG cells the expression of *α*-Sma, a distinctive marker of this phenotype. As Figure 2B and C shows, RMF-EG cells co-cultured with MCF-7 mammary cells express almost four-fold NOX4 mRNA compared with untreated cells. The MCF-7 stimulus enhanced two-fold the *α*-Sma expression, suggesting that the postulated epithelial soluble factors may also constitute a stimulus for myofibroblast differentiation. No stimulatory effect was observed in the case of NOX5.

### Targeted NOX4 knockdown with a specific siRNA blocks RMF-EG stimulus on MCF-7 migration

**Figure 3:**
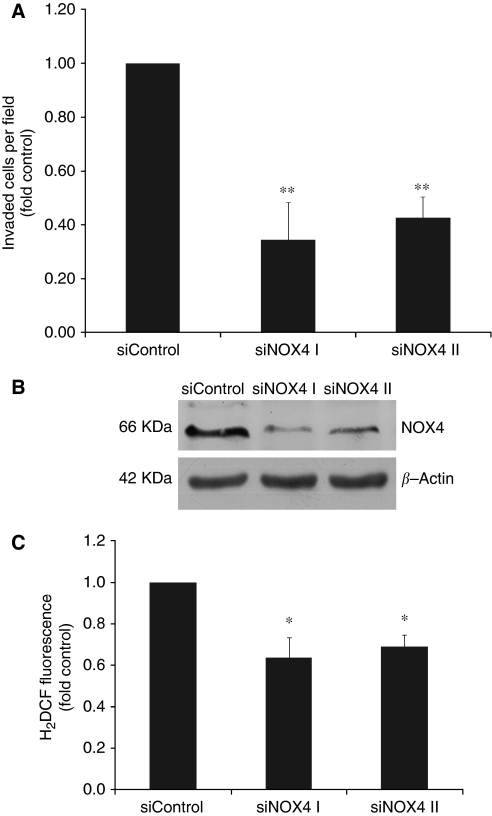
NOX4-targeted knockdown RMF-EG cells do not induce MCF-7 cell migration. Cells transfected with either unspecific siRNA (siControl) or two different specific NOX4 siRNAs (SiNOX4 I and II) were seeded in the lower well of a Transwell double chamber system as in Figure 1 to stimulate MCF-7 cell migration. MCF-7 migration was evaluated as described in the Materials and Methods (**A**). Immunoreactive NOX4 protein expression in transfected cells was evaluated by western blot (**B**). Intracellular ROS production in siRNA- transfected cells was measured with DCDHF-DA as indicated in the Materials and Methods (**C**). ^*^P<0.05 ^**^P<0.01.

To study the possible role of NOX4 in the RMF-EG-mediated stimulus on MCF-7 migration, we used a targeted knockdown by siRNA approach to downregulate the enzyme expression. As Figure 3A shows, RMF-EG cells transiently transfected with siNOX4 were unable to generate a migratory stimulus on MCF-7 cells. We also detected that NOX4 knockdown cells expressed lower levels of NOX4 at the protein level (measured by western blot) and a diminished capacity to generate intracellular ROS (Figure 3B and C).

### Pre-treatment of RMF-EG stromal cells with TGF-*β*1 stimulates cell migration on MCF-7 cells

**Figure 4:**
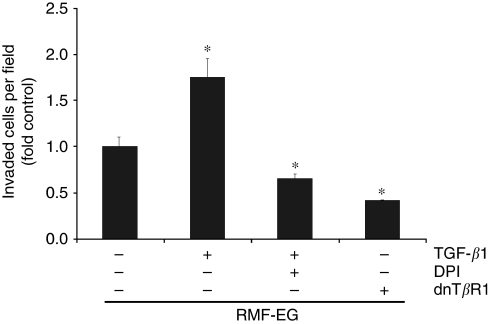
TGF-*β*1-pretreated RMF-EG cells display a greater effect on MCF-7 migration. RMF-EG cells were pre-incubated or not with TGF-*β*1 (10 ng ml^–1^) for 16 h and seeded in the lower well of a Transwell double chamber system as in Figure 1. A group of these factor-treated cells were also incubated with 5 *μ*M diphenyleneiodonium chloride (DPI). Another group of cells were transiently transfected with a dominant-negative version of the type I receptor of TGF-*β*1 (T*β*RI). These cell monolayers stimulated for 24 h the migration of MCF-7 cells seeded in the insert of the Transwell. ^*^P<0.05.

Searching for a specific factor that regulates the RMF-EG-mediated stimulus on MCF-7 migration, we tested whether TGF-*β*1, a growth factor implicated in the control and expression of NOX in some cells (Murillo *et al*, 2007), was able to modulate the NOX4-dependent stimulus of RMF-EG cells. To test this hypothesis, RMF-EG cells were stimulated overnight with TGF-*β*1 (10 ng ml^–1^), previous to the onset of the cell migration assay. As Figure 4 shows, TGF-*β*1 pre-treated RMF-EG cells exhibit almost double effectiveness to stimulate MCF-7 migration. To test if a NOX activity is involved in the TGF-*β*1-dependent enhancement of epithelial migration, we incubated RMF-EG cells with 5 *μ*M of DPI 30 min before pre-treatment with TGF-*β*1. Figure 4 also shows that DPI strongly inhibited TGF-*β*1 stimulus on MCF-7 migration. It is important to note that neither TGF-*β*1 nor DPI were present during the migration assay. Finally, to test if the signalling route activated by the factor is implicated in this effect, we performed a migratory assay using RMF-EG cells expressing a dominant-negative version of the T*β*R1. As Figure 4 shows, these mutant cells are unable to stimulate MCF-7 migration, indicating that the integrity of the TGF-*β*1-dependent signalling route is a requisite for the stimulus on MCF-7 migration.

### TGF-*β*1 stimulates expression of NOX4 in RMF-EG stromal cells

**Figure 5:**
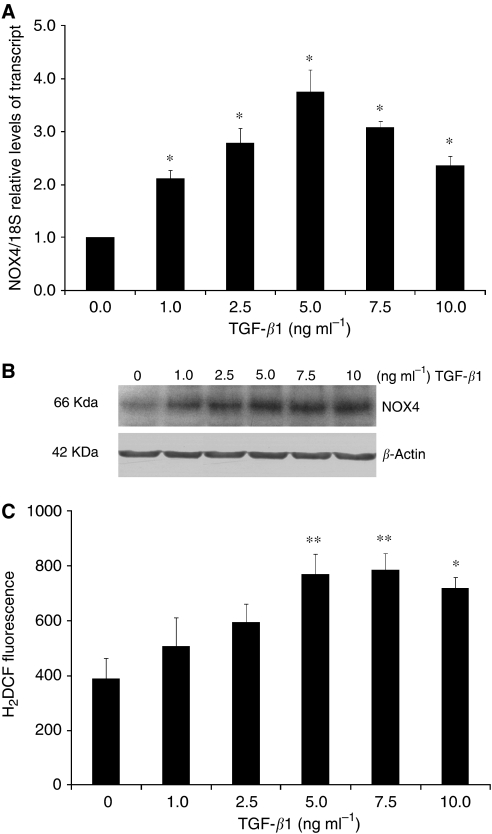
TGF-*β*1 stimulates the expression of NOX4 in RMF-EG cells. Stromal RMF-EG cells were grown until semiconfluence and deprived of FBS overnight. After this, cells were treated with increasing concentrations of TGF-*β*1 for 24 h. Thereafter, a group of cells were used to analyse the mRNA expression through qPCR (**A**), protein expression through western blot (**B**) and intracellular ROS production measuring fluorescence generated after H_2_DCFDA incubation according to the Materials and Methods section (**C**). ^*^P<0.05 ^**^P<0.01.

To analyse whether the effects of stromal NOX4 and TGF-*β*1 activity stimulating MCF-7 cell migration are functionally linked, we studied the effect of TGF-*β*1 on NOX4 expression in RMF-EG mammary stromal cells. To do this, we incubated RMF-EG cells for 24 h with different concentrations of TGF-*β*1 and evaluated the NOX4 expression by real-time PCR. As shown in Figure 5A, TGF-*β*1 exerts a dose-response stimulus on NOX4 mRNA expression in the whole range of concentrations evaluated. The same treatment induces a linear enhancement on NOX4 protein and ROS production, evaluated by measuring the intracellular ROS content by cell fluorescence after loading with DCDHF-DA (Figure 5B and C).

### TGF-*β*1 present in medium conditioned by mammary tumoral cells is responsible for the expression of NOX4

**Figure 6:**
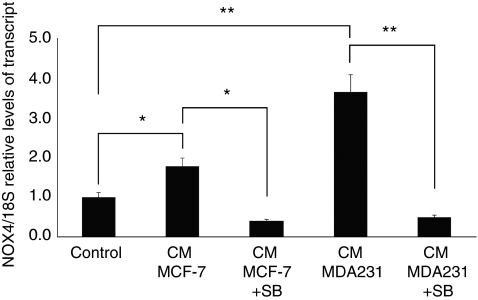
TGF-*β*1 present in medium conditioned by mammary tumour cells is responsible for the upregulation of NOX4 expression in RMF-EG cells. Serum-deprived stromal RMF-EG cells were incubated for 24 h with medium conditioned by MCF-7 and MDA-231 cells in the presence or absence of 10 *μ*M SB431542. NOX4 mRNA expression was evaluated through qPCR as described in the Materials and Methods. ^*^P<0.05, ^**^P<0.01.

To analyse whether TGF-*β*1, a component of the medium conditioned by mammary cells, is responsible of the enhanced expression of NOX4 in RMF-EG cells, we stimulated NOX4 expression by culturing stromal cells with medium conditioned (CM) by weakly (MCF-7) and strongly invasive (MDA-MB 231) human mammary cell lines that express and secrete a level of TGF-*β*1 proportional with their invasive potential, as we have recently demonstrated (Guerrero *et al*, 2010). To identify the contribution of TGF-*β*1 to NOX4 expression, we incubated RMF-EG cells in the presence or the absence of SB431542, a potent inhibitor of T*β*RI kinase activity that causes a blockage of Smad2/3 signalling route (Inman *et al*, 2002). As is shown in Figure 6, the expression of NOX4 mRNA, evaluated by real-time PCR, was stimulated by CMs of mammary cells in a magnitude that is proportional to its invasive potential that, in turn, is directly proportional with their TGF-*β*1 content. SB431542 completely inhibited the stimulus of CMs, suggesting that TGF-*β*1 is the major component of the soluble factors generated by mammary cells that stimulate NOX4 expression.

## Discussion

Cancer is one of the recognised pathologies whose progression has been associated with oxidative stress (Szatrowski and Nathan, 1991). The role of ROS in the initiation, promotion and progression of cancer involves their effects on cell replication, angiogenesis, apoptosis and migration (Halliwell, 2007). Previous data from our group and others have emphasised the importance of the redox balance in the acquisition of a malignant behaviour. These findings confirm the importance of endogenous generation of ROS by members of the NOX family and the regulatory role of the small GTPase Rac 1 in the epithelial migration (Kumar *et al*, 2008; Tobar *et al*, 2008).

Extensive evidence has been generated underscoring the relevance of the stroma in carcinogenesis (Radisky and Radisky, 2007). Recent data using a murine model highlight the importance of the stromal component on mammary tumourigenesis. These researchers have shown that *Pten* genetic ablation in mammary stromal fibroblasts cause ECM remodelling, recruitment of innate immune cells and an acceleration of the initiation, progression and malignant transformation of mammary epithelial tumours (Trimboli *et al*, 2009). In many other examples, tumour cells induce the production of inflammatory mediators that generate a chronic condition that supports the establishment of paracrine signalling that stimulates epithelial tumoral progression (Spaeth *et al*, 2008). Under this persistent inflammatory condition, it is easily expected that the tissue redox balance will be affected mainly by the production of ROS by non-epithelial cells under cancer cell stimulus (Benz and Yau, 2008).

Our results suggest that, under the stimulus of soluble factors secreted by MCF-7 mammary epithelial cells, RMF-EG mammary stromal cells modulate their own oxidative status by increasing the expression of NOX4, an enzymatic complex that has a relevant signalling function in mammary cells (Mahadev *et al*, 2004). Stromal NOX expression was able to stimulate the migratory capacity of MCF-7 cells as we have demonstrated using the siRNA strategy to knock down the expression of NOX4 (Figure 3A–C). These results emphasise the importance of a redox threshold in stromal cells needed to sustain a stimulus for epithelial cell migration. It has been demonstrated that in non-phagocytic cells, NOX4 is predominantly localised to the endoplasmic reticulum where it regulates the activity of protein tyrosine phosphatase 1B (Chen *et al*, 2008). We propose that, as a consequence of NOX4 activity, one (or several) signalling molecule(s) is generated by stromal cells to modulate, in a paracrine manner, epithelial migration. This property seems to be distinctive of tumoral cells because the MCF-10 cell line, which represents a non-tumoral counterpart of MCF-7 cell line, does not respond to the stromal stimulus. Transwell migration experiments performed in MCF-7 cells in the presence of catalase allowed us to rule out the participation of diffusible forms of ROS as part of the migratory stimulus (Figure 1B).

In a previous work, we analysed by immunohistochemistry the presence of TGF-*β*1 in samples of human ductal infiltrant mammary tumours. We found that this factor, which had an important role in the inhibition of the expression of adipogenic transcription factors, was expressed only by tumoral cells located at the invasive front, without a relevant expression in stromal adipose cells (Guerrero *et al*, 2010). In our experimental model, TGF-*β*1 exerts its pro-migratory stimulus through the stromal compartment by a phenomenon that is abolished by pre-treatment with DPI, that is, it depends on NOX activity (Figure 4). Besides this NOX-mediated migration stimulated by TGF-*β*1, the results in Figure 5 also show that NOX4 expression is induced by TGF-*β*1. Therefore, TGF-*β*1 action in this co-culture system may be explained by two simultaneous mechanisms: the NOX-dependent production of a soluble factor that stimulates MCF-7 migration and the enhancement of NOX4 expression by TGF-*β*1. Thus, the generation of a stromal intracellular oxidative environment by epithelial TGF-*β*1 can originate a positive feedback loop that further stimulates the production of soluble factor(s) that favour epithelial cell migration. This finding suggests a new mechanism for tumoral malignancy that may complement the well-known effect of epithelial oxidative environment in the acquisition of cellular migratory capacity (Kumar *et al*, 2008). Furthermore, stromal NOX4 seems to be a molecular target of TGF-*β*1 present in media conditioned by breast carcinoma cell lines, as was confirmed by the use of the T*β*RI inhibitor SB431542 that totally abolished the expression of NOX4 stimulated by media conditioned by MCF-7 and by MDA MB-231 cells, a more invasive cell line that secretes four times more TGF-*β*1 than MCF-7 (Guerrero *et al*, 2010).

TGF-*β*1 has been shown to increase the expression of NOX4 in different types of cells, although the localisation of NOX4 is still under debate. In fetal rat hepatocytes, this growth factor increased NOX4 expression as a part of a factor-induced apoptotic programme (Murillo *et al*, 2007). Also, it has been identified that ROS production in human hepatocyte cell lines previously infected with hepatitis C virus depends on NOX4 activity whose expression is, in turn, stimulated by TGF-*β*1 (Boudreau *et al*, 2009). More recently, it has been demonstrated in murine fibroblasts that the TGF-*β*1-stimulated expression of NOX4 results in the MAPK phosphatase-1 (MKP-1) oxidation, which is directly responsible for the factor-dependent modulation of gene expression (Liu *et al*, 2010). Our current effort is oriented to identify the specific molecular target whose oxidative condition is modified by NOX4, which would be responsible for the stromal stimulus to epithelial migration.

Collectively, the results presented herein emphasise a role for the stroma in the establishment of the patho-physiological conditions that support epithelial malignancy. Our proposal highlights the importance of the generation of an oxidative tumoral environment that favours epithelial motility and contributes, in this manner, to the expression of the malignant phenotype. Stromal NOX4-derived ROS that has been implicated in a variety of physiological processes (Brown and Griendling, 2009) may represent a potential target to prevent the acquisition of malignant properties by the epithelial compartment.
